# Belonging, social connection and non‐clinical care: Experiences of HIV peer support among recently diagnosed people living with HIV in Australia

**DOI:** 10.1111/hsc.13886

**Published:** 2022-06-19

**Authors:** Nathanael Wells, Steven P. Philpot, Dean Murphy, Jeanne Ellard, Chris Howard, John Rule, Christopher Fairley, Garrett Prestage, RISE Study Team, Graham Brown, Jeff Jin, John Kaldor, Rebecca Guy, Andrew Grulich, Limin Mao, Basil Donovan, Asha Persson, Nick Medland, Brent Clifton, Petrina Hilton, Mohamed Hammoud, Suzy Malhotra, Lisa Bastian

**Affiliations:** ^1^ Kirby Institute UNSW Sydney Kensington New South Wales Australia; ^2^ Australian Research Centre in Sex, Health and Society La Trobe University Melbourne Victoria Australia; ^3^ Queensland Positive People (QPP) East Brisbane Queensland Australia; ^4^ National Association of People with HIV Australia Newtown New South Wales Australia; ^5^ Melbourne Sexual Health Centre Melbourne Victoria Australia; ^6^ Central Clinical School Monash University Melbourne Victoria Australia

**Keywords:** HIV, peer support, psychosocial support, quality of life, stigma

## Abstract

Effective HIV treatments have transformed the medical needs of people living with HIV (PLHIV) to a chronic condition. However, stigma, poorer mental health outcomes and social isolation remain significant challenges for many PLHIV. HIV peer support programs have assisted PLHIV in navigating the clinical, emotional and social aspects of living with HIV. We draw on semi‐structured interviews with 26 recently diagnosed PLHIV in Australia to explore experiences of HIV peer support services. Our thematic analysis identified three overarching themes. First, participants commonly reported that peer support programs offered a sense of belonging and connection to a broader HIV community. This established a network, sometimes separate to their existing social networks, of other PLHIV with whom to share experiences of HIV. Second, peer‐based programs provided an opportunity for participants to hear firsthand, non‐clinical perspectives on living with HIV. While participants valued the clinical care they received, the perspectives of peers gave participants insights into how others had managed aspects of living with HIV such as disclosure, sex and relationships. Finally, participants highlighted important considerations around ensuring referrals were made to socially and culturally appropriate support programs. Peer support programs fill an important gap in HIV care, working alongside and extending the work of the clinical management of HIV. Incorporating formal referrals to peer support services as part of the HIV diagnosis process could assist recently diagnosed PLHIV in adjusting to a positive diagnosis.

**What is known about this topic**

- People living with HIV (PLHIV) continue to experience stigma and social isolation.
- Lack of social connection among PLHIV is associated with poorer mental health outcomes (Power et al., 2019).
- Peer‐based programs assist recently diagnosed PLHIV in adjusting to the medical and psychosocial challenges associated with an HIV diagnosis.

**What this paper adds**

- Evidence that peer support programs provide a sense of belonging and connection among recently diagnosed PLHIV and help in adjusting to a positive HIV diagnosis.
- Consideration of gendered, cultural, and/or socio‐demographic needs is important when placing recently diagnosed PLHIV in peer support programs.
- There is a need for clinicians and peer support providers to work closely with recently diagnosed PLHIV to ensure appropriate support is given.

## 1 INTRODUCTION

Effective HIV treatments have transformed the medical needs of people living with HIV (PLHIV). By reducing the virus to undetectable levels, HIV treatments can improve the short‐ and long‐term medical outcomes of PLHIV while also eliminating the risk of sexual transmission (Bavinton et al., 2018; Rodger et al., 2016). For those with access to treatments, HIV is increasingly reframed as a chronic medical condition that can have minimal impact on day‐to‐day life (Moyer, 2015; Moyer & Hardon, 2014; Newman et al., 2015). However, PLHIV often experience increased medical and psychosocial comorbidities that can impact the management of HIV (Bulsara et al., 2019). Alongside the clinical management of HIV, stigma, adverse mental health outcomes and social isolation remain significant challenges for many PLHIV (Grov et al., 2010; Parker & Aggleton, 2003; Perazzo, 2017; Power et al., 2019), and can negatively affect health‐seeking behaviours (Bulsara et al., 2019; Dang et al., 2017).

Access to, and uptake of, treatment is central to the United Nations' 90‐90‐90 targets to have 90% of PLHIV aware of their status, 90% of those aware on treatment and 90% of those on treatment virally suppressed (UNAIDS, 2014) and is key to Australia's National HIV Strategy (Department of Health, 2018). However, access to treatment and reduced mortality do not necessarily equate with improved quality of life (Walker, 2019). Recognising this, Lazarus et al. (2016) called for the addition of a fourth ‘90’ to the United Nations targets: to see 90% of those virally suppressed reporting good health‐related quality of life. Lazarus et al. (2016) draw attention to the importance of HIV care beyond simply the clinical, medical and pharmaceutical management of the virus. Improving the social wellbeing of PLHIV underpins Australia's National HIV Strategy (Department of Health, 2018). This requires tailored and holistic approaches to HIV care that address the medical aspects of HIV and the complex psychological, emotional and social challenges experienced by PLHIV (Flowers et al., 2011; Gunaratnam et al., 2019; Mikołajczak et al., 2021).

For some, reactions to a positive HIV diagnosis can be marked by shock, confusion, isolation and despair (Bilardi et al., 2019; Flowers et al., 2011). Bilardi et al. (2019) demonstrate, however, that when diagnosed those with other PLHIV in their social networks are better able to adjust to a positive diagnosis. Similarly, Power et al. (2019) demonstrate that lack of social connection among PLHIV is strongly associated with negative health‐related quality of life. For those with no PLHIV in their social networks, peer support programs can work alongside and extend the work of HIV clinicians to support PLHIV in navigating the clinical, emotional and social aspects of living with HIV (Khalpey et al., 2021; Mikołajczak et al., 2021; Prestage et al., 2016). Peer support programs can act as a bridge between PLHIV and healthcare systems (Davis et al., 2017; Khalpey et al., 2021; Koester et al., 2014; Ogier et al., 2020), increase treatment uptake, adherence and retention in care (Berg et al., 2021; Cunningham et al., 2018; Waldrop‐Valverde et al., 2014) and decrease HIV risk behaviours (Prestage et al., 2016). These programs can also build resilience among PLHIV to reduce the impact of HIV stigma (Chan & Mak, 2021; Hussen et al., 2021) and establish social connections between PLHIV (Davis et al., 2017; Khalpey et al., 2021). Peers are often members of the same communities as those they support, have experiential knowledge of living with HIV (Berg et al., 2021; Ogier et al., 2020; Peterson et al., 2012), and can offer recently diagnosed PLHIV unique perspectives that clinically based healthcare workers are often unable to provide (Barker & Maguire, 2017; Davis et al., 2017; Fisher et al., 2018; Koester et al., 2014; Newman et al., 2015; Peterson et al., 2012).

While significant attention has been given to the impact of peer‐based programs on HIV clinical care, little attention has been given to how these programs are experienced by recently diagnosed PLHIV. Drawing on semi‐structured interviews with recently diagnosed PLHIV, this paper provides important insights for both clinicians and community‐based HIV organisations when considering referrals to, and development of, peer support programs. In this analysis, we describe: peer support as providing and sense of belonging to a broader HIV community; the role of peer navigators in providing a lived experience perspective and, considerations for placing recently diagnosed PLHIV in appropriate peer programs.

## 2 METHODS

### 2.1 Study setting

The RISE study is an ongoing cohort study of recently diagnosed PLHIV in Australia. Participants were initially recruited to complete a survey every 6 months and were given an option to participate in an in‐depth interview, including a follow‐up interview 12 months later. As the study progressed the survey component was discontinued, and participants could enrol directly into the interview component. This paper is based on data from interviews conducted between January 2019 and February 2021. Ethics was approved by the UNSW Human Research Ethics Committee.

### 2.2 Eligibility and recruitment

Eligible participants were 16 years or older, a permanent or temporary Australian resident, and diagnosed in 2016 or later. Participants were recruited through community‐based HIV organisations, sexual health centres, clinicians, and/or self‐referral. Potential participants were contacted via telephone or email by a member of the research team and invited to be interviewed.

### 2.3 Data collection

In‐depth, semi‐structured interviews were conducted face‐to‐face, via telephone, or through video conferencing by SP and DM. Interviews were audio recorded with participants' consent, transcribed by a professional transcription service and de‐identified. Interviews generally took between 90 and 120 minutes and covered: the experience of diagnosis; HIV treatment and clinical care; engagement with HIV support services (including peer support), sex and relationships, likely source of infection and the overall impact of HIV on participants' lives. Follow‐up interviews, conducted approximately 12 months after the initial interview, explored any shifts in participants' experiences of living with HIV.

### 2.4 Analysis

Interviews were entered into NVivo software version 12 and thematically analysed (Braun & Clarke, 2006). An initial codebook was developed by SP and DM based on a close reading of a small sample of interviews and subsequent discussion with members of the research team. These codes were applied to the remaining data and the codebook revised as additional transcripts were coded. Analysis for this paper was primarily conducted by NW. Members of the research team met regularly to review and discuss further findings as they arose.

## 3 RESULTS

Of the 26 participants who completed first‐round interviews, approximately three quarters (19) completed follow‐up interviews after 12 months. Participants received a positive HIV diagnosis between February 2016 and March 2020. Most participants identified as gay men (15), five as bisexual men, four as heterosexual men and two as heterosexual women. Participants were born in Australia/New Zealand (19), Europe (3), South America (2), Asia (1) and Africa (1). Participant characteristics are described in Table 1. Except for one participant not on treatment, all participants had an undetectable viral load at their first interview.

**TABLE 1:** Participant characteristics

Age	
20–29	11
30–39	6
40–49	5
50–59	4
Gender	
Male	24
Female	2
Sexuality	
Gay male	15
Bisexual male	5
Heterosexual male	4
Heterosexual female	2
Region of birth	
Australia/New Zealand	18
Indigenous Australian	1
Europe	3
South America	2
Asia	1
Africa	1
Year of HIV diagnosis	
2016	5
2017	8
2018	5
2019	6

All participants were aware of community‐based peer support services and programs for recently diagnosed PLHIV. Of the 26 participants in this analysis, 20 reported having engaged with peer support services and/or programs. These included one‐on‐one peer navigation services and formal workshops. Workshops, usually held over 2 days, included structured education around aspects of living with HIV and facilitated social connection with other PLHIV. Participants also described attending less formal social groups run by community‐based HIV organisations and joining the Australian‐run Facebook group TIM (The Institute of Many), a digital support network for PLHIV. TIM is a moderated, independent Facebook group and is only open to PLHIV. While TIM's membership is primarily Australian based, it also includes members from outside Australia.

### 3.1 Theme 1: Peer‐based programs as providing interpersonal and social support

Participants commonly described how peer support programs facilitated interpersonal and social connection with other PLHIV. This diminished feelings of isolation and also provided an opportunity to hear the experiences of other PLHIV. Cameron (gay male, 30) explained his reasons for attending a group workshop for recently diagnosed PLHIV as:

not because I really needed to know anything else [about HIV]. I just needed to meet other people. I was just going crazy and I did not really know anywhere else. I just needed a jumping‐off point … It was just nice to have a whole group of other guys in the same position … It was just nice to just hang out.

Cameron's primary motivation for attending a workshop was not to increase his awareness of HIV nor to learn from the experiences of other PLHIV. Rather, his lack of social interaction with other PLHIV had negatively impacted Cameron's mental and emotional wellbeing. Unsure of where else to go, formal peer workshops acted as a ‘jumping‐off point’, an avenue through which Cameron could begin to connect socially with others in a similar position to himself.

Some participants commented that simply hearing how other PLHIV responded to their diagnoses normalised their own strong emotional reactions. At the time of his diagnosis, Rhys (gay male, 35) had recently migrated to Australia and did not have an established social network. Describing his experience of a group workshop, Rhys stated:

I heard people that had the same fear as I did. It wasn't just me being frightened, and ignorant, and stupid, and feeling inadequate. It was all these people from different walks of life. All these guys sitting there and just being very vulnerable and open.

Rhys' reaction to his HIV diagnosis was marked by strong negative emotions that went beyond concern about the medical impact of HIV and extended to measuring his own self‐worth. By hearing the experiences of others experiencing similar emotions, Rhys felt validated and came to view his own reactions as unexceptional. Rhys continued:

I generally find that quite repulsive, … listening or gushing about my emotions … I really enjoyed it to my own surprise. It was refreshing to be able to talk about this … Most of my friends that I'd made did not know that I was HIV‐positive, and it was a relief, really. It was a way for me to express things and I felt fairly unjudged in that sense. Everyone had weird, sad stories and it was okay.

Despite his initial reservations about the group format and sharing his emotions, hearing from others created a space in which Rhys felt comfortable sharing his own challenges. Feeling unable to discuss his diagnosis with others in his social networks, the group workshop was an outlet through which Rhys could work through the negative emotions he was experiencing with others who understood his situation. Through participating in the peer group, Rhys discussed his experiences of living with HIV without fear of judgement and described a sense of ‘relief’ in doing so.

The sense of interpersonal and social support was not limited to in‐person, formal peer support but also extended to digital networks such as TIM. Angus (gay male, 28) stated:

I'd scroll through the member list and see all the different members in the group, and you'd see people who were just like you. It was good to feel part of a majority again and not feel like it was just you that was going through this … And it was kind of like, ‘okay, this is good. I won't talk, I'm not going to reach out to them or anything like that.’ But it was just reassuring to see that you are not alone.

Despite not actively participating in group discussions, Angus' membership in the group diminished his sense of social isolation. Although Angus felt a sense of support through TIM, others described negative interactions as deterring them from actively participating in the group. Zaid (gay male, 40) explained:

Nine times out of 10 whenever I'd look at TIM, the reason I would not continue reading is because there's a nasty commentary on there and I was just trying to make sure I did not have any bullshit in my head. Whenever there was the [negative] type comment, I would just not continue on.

While the negative conversations described here were often associated with social media itself and not unique to TIM, they did discourage some participants from fully engaging with the group. Despite the negative comments, however, Zaid ultimately believed TIM was an important resource, stating:

I think it's really, really good. I think it's brilliant … It's just good every now and again to check in and just see peoples' commentary and see how they are managing their head with the diagnosis.

Despite the sometimes‐negative commentary occurring on TIM, Zaid considered TIM a valuable way of maintaining some connection with other PLHIV. Like Angus, Zaid did not actively participate in discussion on TIM's social media page but rather, Zaid felt a tacit sense of support simply through observing other group members.

### 3.2 Theme 2: Peer navigators as providing a lived experience perspective

A benefit of peer support—particularly one‐on‐one peer navigation—described by some participants was that it offered non‐clinical, firsthand perspectives on living with HIV. While participants generally trusted the health advice of their HIV clinicians, peer navigators were able to offer firsthand perspectives on the day‐to‐day experience of living with HIV. Zaid (gay male, 40) described his motivation for participating in a peer navigation program:

I just wanted a person who's going to say “Right, this is what happens. This is what you're going to do. This is what you're not going to do.” … He [peer navigator] reiterated from a life perspective, from a living perspective, exactly what they [clinicians] had said … Sometimes you need a lived perspective. You know, what it is actually like to do something [while living with HIV].

Zaid trusted the expertise of his HIV clinician and valued their relationship. However, he also felt a need for support beyond the clinical encounter. Zaid noted that the peer navigator effectively restated what had already been conveyed by his clinician. By drawing on their own lived experience, however, the peer navigator was well situated to play a translational role by conveying clinical information from a different, lived experience perspective.

Unlike the clinical context, in which there is an inherent power imbalance between the clinician and patient, the relationships between peer navigators and recently diagnosed PLHIV are generally less formal and more equal (Berg et al., 2021). By drawing on their own experiences as examples, peer navigators can also act as demonstrative that a positive HIV diagnosis need not necessarily have a negative impact on their future. Diego (bisexual male, 33) explained:

[it] was a good experience because he said: ‘okay, I got [HIV] before my kids. And my kids and my wife, they do not have [HIV]. So, I felt really good when I hear[d] that because I probably want to have a family. I want to have some kids.

For Diego, hearing these experiences instilled a sense of optimism about his future possibility of raising children and starting a family. While these experiences did not necessarily diminish the immediate challenges for Diego in adjusting to his diagnosis, speaking with a navigator normalised living with HIV, demonstrating that these challenges could be overcome. This was reflected by Percy (gay male, 26), who stated:

I was talking to someone else who was living with HIV who wasn't necessarily what you typically see in the media as, like, a victim. It was more, they were an empowered young person. This is someone who is similar to me, who is living [and] getting on with life.

Percy's interaction with a peer navigator challenged his notion of what a ‘typical’ person living with HIV was like. Rather than a passive ‘victim’, Percy described his peer navigator as ‘empowered’ and as someone he could relate to. In this way, peer navigation can also work to disrupt the impact of perceived and/or internalised HIV‐related stigma, with peer navigators acting as role models of living a fulfilling life with HIV.

For some participants, one of the benefits of peer support programs was their scope to address social challenges experienced by PLHIV. Speaking of his experiences in a group workshop, Ronny (gay male, 42) commented:

It spoke directly to disclosure, it spoke directly to getting back into the dating scene, it spoke directly to health. It spoke directly to all of those types of things … The disclosure part was very beneficial.

In contrast to medical contexts, a key focus of peer support programs is addressing, through first‐hand experiences, social aspects of living with HIV such as disclosure and navigating sexual and/or romantic relationships. These aspects of HIV care are often outside the scope of what clinicians can provide in terms of both expertise and resources (Newman et al., 2015) and fill an important gap in clinical care.

### 3.3 Theme 3: Considerations when matching PLHIV to appropriate support

While participants generally described positive encounters associated with peer support, there were some reports of negative experiences. Most commonly, this was due to a perceived mismatch when participants were placed with peer navigators or peer support groups. Dexter (gay male, 50) explained:

I had real problems with [the peer workshop] in the beginning … because the group that I was placed into was a mature‐age, long‐term survivor group. And I'm not a long‐term survivor, I'm dealing with day one issues … I could not discuss anything with this group. I could not get any of the conversations that were going to be effective for me in this group because the group had been there and done that 20 years ago. And so they were actually quite blasé about it, and so they could not understand that this was actually still a really real concern for me … So, actually talking to someone who was 29 and recently diagnosed was actually more effective for me.

When placed in a support group, Dexter believed the primary consideration had been his age rather than his recent HIV diagnosis and so was placed accordingly. While the group did provide some social connection, this placement led to Dexter feeling that his concerns about his recent diagnosis were not taken seriously. Dexter ultimately sought out the support of other recently diagnosed PLHIV and described feeling more supported by someone almost 20 years his junior. For Dexter, it was the shared experience of a recent HIV diagnosis, not a shared age demographic, that offered him the most appropriate support, highlighting the importance of considering how ‘peer’ is defined in the context of HIV peer support programs.

In contrast to Dexter, whose primary concern was a shared experience of recent HIV diagnosis, other participants described shared experiences of cultural background and gender as important when engaging with peer support programs. Amber (heterosexual female, 29) was involved in a program open to all PLHIV regardless of gender and/or sexual identity. Through this, she was exposed to a broad range of different experiences and, like Dexter, valued hearing the perspectives of other recently diagnosed PLHIV. However, Amber also emphasised the importance of shared experience beyond just HIV status, stating:

There are a few other heterosexual women that do attend … We have big discussions on how they approach situations, how they approach disclosing to a new partner, or how they have decided to disclose to their children. Or even just the fact that some of our medication … that guys do really quite well on are some medications that we cannot take purely because of the pill.

For Amber, having other heterosexual, female PLHIV provided an opportunity to discuss and consider the impacts of HIV on aspects such as reproductive health. As described previously, this was important in providing firsthand, non‐clinical aspects on the experiences and challenges others had encountered.

Bunji (gay male, 41) emphasised the importance of engaging with peer support programs that were culturally appropriate, particularly for those who experience multiple and intersecting forms of marginalisation. Indigenous Australian himself, Bunji reflected on his experience of peer navigation:

When I got in contact with [HIV organisation] to speak to someone that had this condition, they gave me a person to talk to, and they gave me a white, middle‐aged woman from the suburbs.

Later in the interview, Bunji continued:

Why aren't there peers for Aboriginal and peers for Torres Strait [peoples]? Why are they giving us anyone to work with? We have to explain to them our life, our lived experience, [and] our trauma before they can try and give us some insight into disclosure, which they are no good at because they would never understand.

Bunji described having to go through an extra process of explaining to peer navigators how factors such as intergenerational trauma, colonisation and historical and continued institutional racism impact Indigenous Australians' interactions with healthcare systems (Negin et al., 2015; Ward et al., 2018). Even after explaining these issues, however, Bunji felt peer navigators without these shared experiences were unable to offer adequate support. While the shared experience of HIV was an important aspect of peer support for both Amber and Bunji, it was also felt important for support programs to include shared experiences around gender and cultural background.

## 4 DISCUSSION

Our analysis highlights the important role of peer support for recently diagnosed PLHIV when adjusting to an HIV‐positive diagnosis. Participants commonly described how, working alongside the clinical management of HIV, peer‐based programs offered support in adjusting to the social, emotional, clinical and psychological challenges associated with an HIV diagnosis. Among our sample, social connection with other PLHIV was valued as an important aspect of peer support services. Previous research has shown that an HIV diagnosis is often associated with feelings of shame, fear of rejection, withdrawal from social life and loss of social connection with friends, family and romantic/sexual partners (Hollingdrake et al., 2017; Power et al., 2021). Social connection plays an important role in adjusting to a positive HIV diagnosis and is associated with improved mental health, well‐being and quality of life (Brener et al., 2020; Kendall & Rogers, 2007; Lyons & Heywood, 2016; Power et al., 2019). Social and community connection can also mitigate against the negative effects of HIV stigma (Brener et al., 2020; Chan & Mak, 2021). By fostering social connection among PLHIV, peer support services fill an important gap in the clinical management of HIV care.

Our findings reflect previous research that demonstrates peer‐based programs can support PLHIV in navigating the clinical, social and emotional aspects of living with HIV (Cabral et al., 2018; Khalpey et al., 2021; Koester et al., 2014; Peterson et al., 2012). Participants frequently described how peer support programs were an opportunity to hear firsthand accounts of how other PLHIV managed non‐clinical aspects such as disclosure and relationships. Reactions to a positive HIV diagnosis are often marked by uncertainty, confusion and concern for the future. Aligning with the findings of Koester et al. (2014), participants in our study also described peer navigators as demonstrating, through their own lived experience, that HIV need not be a barrier to achieving many of the life goals held prior to diagnosis. This highlights the importance of linking PLHIV to peer navigation programs as soon after diagnosis as possible.

Despite participants reporting mostly positive experiences with peer support, our findings highlight important considerations when referring recently diagnosed PLHIV to appropriate peer support programs. Quinn et al. (2018) emphasise the need to consider ‘intersectional stigma’ when delivering HIV peer support, particularly for those who also experience marginalisation around racial, sexual and gender identities. While for some participants the shared experience of recent diagnosis was of primary importance, others highlighted the need for peers who also had similar life experiences beyond HIV. Given this, it is necessary to consider how ‘peer’ is deployed beyond shared HIV status, and also encompasses multiple and intersecting social demographics. One of the significant challenges for community‐based organisations is meeting the diverse needs of individual PLHIV within the constraints of often limited resources and funding.

While peer support is recognised by HIV clinicians as an important part of HIV care (Khalpey et al., 2021), referral pathways to these services are often unstructured and informal (Murphy et al., 2017). Linking recently diagnosed PLHIV to peer support services immediately after diagnosis can provide an important source of emotional support (Davis et al., 2017). Given this, it is important that referrals to HIV peer organisations occur at the initial diagnosis to best ascertain the most appropriate programs for those recently diagnosed. As adjusting to an HIV diagnosis is not a linear process (Hollingdrake et al., 2017), PLHIV may also benefit from peer support services, such as TIM, that allow for engagement when needed and at multiple points throughout the life trajectory of living with HIV. Given the complex and various needs of PLHIV, it is necessary for clinicians and peer support providers to work closely together to build referral pathways, relationships and shared models of care to best support PLHIV.

### Limitations

Our analysis is primarily focused on the experiences of those who accessed HIV peer support through established organisations and does not consider the informal peer networks of participants. As informal peer networks also play an important role in supporting PLHIV, consideration of both informal and formal support networks is warranted. Our analysis was also unable to measure the impact of peer support programs on the health‐related quality of life of participants, nor compare this with those who did not access peer‐based programs. While measuring the impact of specific programs is important, our analysis sought an in‐depth understanding of *how* these programs were experienced by participants. Similarly, conclusions around the gendered and cultural appropriateness of programs are taken from a relatively small number of participants. Finally, potential participants were referred through HIV clinicians and community‐based HIV organisations and, except for one, all participants were engaged in some form of HIV care at the time of their interview. Our sample is therefore not representative of those who are disengaged with HIV clinical care. Future analyses should consider why people choose to not access peer programs, barriers to programs among highly vulnerable PLHIV and the impact of these programs on overall health‐related quality of life.

## 5 CONCLUSION

Peer support programs fill an important gap in HIV care, working alongside and extending the work of the clinical management of HIV. Peer support can offer a sense of social connection and belonging to a broader HIV community. Peer programs also provide an opportunity for recently diagnosed PLHIV to hear first‐hand accounts of how others adjusted to living with HIV. PLHIV have diverse needs and identities so careful consideration of culturally and socially appropriate support is necessary when working with recently diagnosed PLHIV. It is important for clinicians and peer support providers to work closely with individuals to best understand their unique needs, while also leveraging their own expertise, to ensure recently diagnosed PLHIV are matched with the most appropriate peer support. Incorporating formal referrals to peer support as part of the diagnosis process could assist recently diagnosed PLHIV in adjusting to a positive diagnosis, while also providing social and peer connection, and non‐clinical perspectives on living with HIV. At the same time, it is vital that these services be adequately resourced to best meet the complex, diverse and multiple needs of all PLHIV.

## AUTHOR CONTRIBUTIONS

Conceptualisation: Garrett Prestage; Steven Philpot; Dean Murphy. Methodology & Data Collection: Garrett Prestage; Steven Philpot; Dean Murphy; Nathanael Wells. Formal analysis and investigation: Nathanael Wells; Steven Philpot; Dean Murphy; Garrett Prestage. Writing – original draft preparation: Nathanael Wells. Writing – review and editing: Nathanael Wells; Steven Philpot; Garrett Prestage; Dean Murphy; Jeanne Ellard; Chris Howard; John Rule; Christopher Fairley.

## FUNDING INFORMATION

The RISE Study is funded by the National Health and Medical Research Council (NHMRC) and the health departments of the Australian Capital Territory, New South Wales, Northern Territory, Queensland, South Australia, Tasmania, Victoria and Western Australia.

## CONFLICT OF INTEREST

All authors declare no conflict of interest.

## CODE AVAILABILITY

Codes were generated using NVivo software v.12.

## ETHICS STATEMENT

All procedures involving human participants were in accordance with the ethical standards of the institutional research committee and with the 1964 Helsinki declaration and its later amendments or comparable ethical standards. Ethics approval was provided by the UNSW Human Research Ethics Committee (HC1804590).

## CONSENT TO PARTICIPATE

Informed consent was obtained from all individual participants included in the study.

## CONSENT FOR PUBLICATION

N/A.

## Data Availability

The data that support the findings of this study are available on request from the corresponding author. The data are not publicly available due to privacy or ethical restrictions.

## RISE Study Team

*Chief and associate Investigators*: Garrett Prestage, Graham Brown, Jeff Jin, John Kaldor, Rebecca Guy, Andrew Grulich, Limin Mao, Basil Donovan, Asha Persson, Nick Medland, Christopher Fairley.

*Project staff*: Brent Clifton, Dean Murphy, Steve Philpot, Petrina Hilton, Mohamed Hammoud, Nathanael Wells.

*Advisory Committee*: Garrett Prestage, Graham Brown, Dean Murphy, Jeanne Ellard, John Rule, Suzy Malhotra, Chris Howard, Lisa Bastian, Steve Philpot, Brent Clifton.

*Partnership Organisations*: ACON, Australian Federation of AIDS Organisations (AFAO), Australasian Society for HIV Medicine (ASHM), Meridian (formerly AIDS Action Council ACT), Northern Territory AIDS and Hepatitis Council (NTAHC), National Association of People with HIV Australia (NAPWHA), Positive Life NSW, Living Positive Victoria, Queensland Positive People, Sexual Health Information Networking & Education SA (SHINE SA), Tasmanian Council on Aids, Hepatitis and Related Diseases (TasCARHD), Western Australian AIDS Council (WAAC), ACT Health, Department of Health Western Australia, Department of Health and Ageing South Australia, Department of Health and Human Services Tasmania, NSW Ministry of Health, Northern Territory Department of Health, Queensland Health, Victorian Department of Health and Human Services.
